# Experimental investigation on strengthening of Zea mays root fibres for biodegradable composite materials using potassium permanganate treatment

**DOI:** 10.1038/s41598-024-58913-y

**Published:** 2024-06-04

**Authors:** S. Anne Kavitha, Retnam Krishna Priya, Krishna Prakash Arunachalam, Siva Avudaiappan, Erick I. Saavedra Flores, David Blanco

**Affiliations:** 1 PG & Research Department of Physics, Holy Cross College (Autonomous), Nagercoil, Manonmaniam Sundaranar University, Tirunelveli, 627012 India; 2 Departamento de Ciencias de la Construccion, Facultad de Ciencias de la Construccion y Ordenamiento Territorial, Universidad Tecnologica Metropolitana, Dieciocho 161, Santiago, Chile; 3 Departamento de Ingeniería en Obras Civiles, Universidad de Santiago de Chile, Av. Ecuador 3659, Estación Central, 9170022 Santiago, Chile

**Keywords:** Zea mays, Potassium permanganate, Crystalline, Physical analysis, Thermal stability, Engineering, Materials science

## Abstract

Humans are the only species who generate waste materials that cannot be broken down by natural processes. The ideal solution to this waste problem would be to employ only compostable materials. Biodegradable materials play a key role in creating a safer and greener world. Biodegradability is the gift that keeps on giving, in the sense of creating an Earth worth living. The future is thus best served by green energy, sustainability, and renewable resources. To realize such goals, waste should be considered as a valuable resource. In this context, *Zea mays* (Zm) root fibres, which are normally considered as agricultural waste, can be used as reinforcing substances in polymer matrices to produce structural composite materials. Before being used in composites, such fibres must be analysed for their physical properties. Chemical treatments can be employed to improve the structural quality of fibres, and the changes due to such modification can be analysed. Therefore, the current work examines the effect of permanganate treatment on the surface properties of Zm fibres. The raw and potassium permanganate-treated samples were assayed for various properties. Physical analysis of the fibre samples yielded details concerning the physical aspects of the fibres. The thermal conductivity and moisture absorption behaviour of the samples were analysed. Chemical analysis was employed to characterize the composition of both treated and untreated samples. p-XRD was employed to examine the crystalline nature of the Zm fibres. Numerous functional groups present in each sample were analysed by FTIR. Thermogravimetric analysis was used to determine the thermal stability of Zm fibres. Elemental analysis (CHNS and EDS) was used to determine the elemental concentrations of both raw and treated samples. The surface alterations of Zm fibres brought on by treatment were described using SEM analysis. The characteristics of Zm roots and the changes in quality due to treatment were reviewed, and there were noticeable effects due to the treatment. Both samples would have applications in various fields, and each could be used as a potential reinforcing material in the production of efficient bio-composites.

## Introduction

Humans cause the majority of environmental damage as a result of their ignorance. Recent environmental issues such as global warming present a challenge to the global community. This is primarily caused by people's urban lifestyle. The usage of synthetic and plastic materials is one of the main contributors to this problem. Plastics are in a sense more lethal than nuclear weapons, and future generations will be affected by the effects of plastic waste for decades. Therefore, there must be a major effort made to limit the use of plastics. Even the world's most powerful countries are unable to completely eradicate pollution on their own; instead, they must rely on the choices of ordinary people and their will to make changes. Whether a person is willing to make a sacrifice now for the benefit of future generations whose appreciation will not be observed is the ultimate moral test of such an individual. At all times, one must embody change if one wishes to see progress made. Pollution can be avoided by choosing renewable resources to create sustainable products. In order to improve those products and increase customer satisfaction, the physical and mechanical qualities of materials must be investigated. Polymer composites are one such class of materials, providing higher productivity, cheaper costs, and greater ease of processing. The mechanical properties of conventional composites such as carbon or glass fibres are superior to those of natural fibres. However, the production of these fibres requires energy, and thus their continued usage harms living organisms and pollutes the environment.

Natural fibres are simply defined as those that are not artificial. They may originate from either flora or fauna^1^. High abundance, excellent specific strength, inexhaustibility, relatively low density, rigidity, enhanced corrosion resistance, and being less aggressive to machinery are some of the advantages that natural fibres have over man-made fibres. In addition to these benefits, they are biodegradable and can be buried in the ground once they have served their purpose. In this work, the hydroponically grown *Zea mays* root fibres, which are often disposed of as undesired waste after the grass is used as animal fodder, may be considered a recyclable substitute for usage in polymeric composite materials. A step towards sustainable development is the use of agricultural waste as a natural fibre. India, a predominantly agricultural nation, has enormous potential for using agricultural wastes in natural fibre-reinforced composites^2–6^.

Composites primarily consist of two components. There could be more than two components in a composite system. The main portion of composites that has a continuous phase and comprises most of the composite is referred to as the matrix. Typically, the matrix is more ductile and less rigid. Both organic and inorganic matrices are common. The secondary constituent is a matrix-embedded discontinuous phase material. Reinforcement describes the interaction from this combination of materials. Although the components of composites have their distinctive chemical and physical characteristics, after being combined, they form a set of characteristics that any one of the constituents alone would not be able to achieve^7^.

Depending on how they are used, natural fibres are frequently separated into primary and secondary types. Sisal, hemp, jute, and kenaf fibres, which are obtained from plants grown specifically for their fibre content, are examples of primary fibres. Agro leftovers, PALF, and coconut fibres, which are by-products of plant products, are examples of secondary fibres. These fibres can be further divided into six categories, including leaf fibres (pineapple leaf fibre, sisal), bast fibres (jute, flax), seed fibres (coir, cotton), reed and grass fibres (maize, rice, wheat), core fibres (hemp, kenaf) and all other kinds (roots and wood)^8–10^.

Despite having several benefits, natural fibres have some disadvantages when used in polymer matrix composites. Their hydrophilicity, excessive moisture absorption, inadequate resilience to high temperatures, unpredictability in fibre characteristics, and fragile bonding with the hydrophobic polymeric matrices are some of these drawbacks. Therefore, it is necessary to enhance natural fibres' physical and chemical characteristics in order to create a strong bond with the matrix. Treatment methods for lignocellulosic materials include chemical (alkali, benzoylation, potassium permanganate, silane, acetylation, and stearic acid treatments), physical (ozone, laser, mechanically induced surface fibrillation, and plasma treatments), and microbiological methods. Each technique has its own benefits and drawbacks. Depending on the source of the material and the goal of the process, the efficiency and efficacy of its utilisation may differ. The most popular technique for treatment is chemical since it is simpler, more efficient, quicker, and requires less energy^11–13^.

The pre-treatment of the fibre surface is an option for surface modification. The preferred pre-treatment uses alkali, which dissolves OH groups and eliminates amorphous contents and oils from the fibres, thereby improving their mechanical qualities. It has been demonstrated to be quite effective to apply KMnO_4_ to the surfaces of natural fibres in order to modify them, and this increases chemical interlocking at the interface. Although prolonged soaking in high concentrations of KMnO_4_ and NaOH can cause the separation of fibre bundles into individual fibrils, this can also result in the elimination of fibre components and a reduction in tensile strength. Understanding the structural properties of a natural fibre is crucial in determining its suitability for its usage in various applications, making it vital to comprehend the structural alterations brought on by using chemicals during fibre modification. In order to use Zm root fibres as reinforcing materials in structural materials, a thorough investigation of the fibre's physical, chemical, and morphological properties is necessary^14,15^.

A sustainable future in architecture is demonstrated by natural fibres that can be utilised to produce a variety of structures and objects as well as to improve currently popular materials^16–19^. Furniture, railroad ties, automobile frames and carpeting, horticultural supplies, packing material, and essentially any use that does not demand a high level of mechanical tolerance but instead requires affordable purchase and low maintenance are examples of already-explored industrial applications. Additionally, swapping out raw polymers with recycled material is easy and practical, improving both economic effectiveness and environmental sustainability^20–23^.

The quality of fibres also depends on the fibre's length, age, and separation methods. Low density and decreased mass fraction are the two main factors that favour the use of natural fibres as reinforcements. The structural elements of vehicles must be made of a lightweight material to reduce their energy consumption. With their capacity to bend to a variety of designer shapes, natural fibre composites are now setting new trends. The use of composites with natural fibre reinforced polymers has grown during the last few years in various industrial areas^24–27^. The global market for organic fibre-reinforced polymer composites is predicted to continue to develop^28,29^. Zea mays roots grown by hydroponic method is a novel natural fibre to be investigated in this field. All the characteristics of this fibre material are studied using various investigations. One remarkable quality is that the density of raw Zm fibres are low compared to many other natural fibres.

## Materials

The maize seeds needed for the growth of corn grass were obtained from a neighbourhood store at Panagudi, Tirunelveli District, Tamil Nadu, India. Sodium hydroxide pellets, KMnO_4_ salt, and acetone were the chemicals employed in this study and were purchased from Spectrum Reagents and Chemicals Pvt. Ltd., Cochin. For the chemical processing of root fibres, double-distilled water from the research lab of the Department of Physics at Holy Cross College, Nagercoil, was used.

### Material importance—*Zea mays*

Poaceae, originally known as Gramineae, is the family that includes maize. Maize is categorised botanically as a grass, with the species name *Zea mays*. Maize is the most productive and widely grown crop in the world. It is widely cultivated for its use as a biofuel, as a raw material in manufacturing, and as food for both people and animals. Numerous maize varieties are grown primarily for their industrial uses, and the maize plant itself has many parts that are utilised in several applications. The preparation of industrial solvents as well as the production of charcoal all directly involve the usage of corn cobs as fuel. Corn stalks are employed in making paper and boards. A lengthy tradition of using maize husks in folk art has resulted in items such as corn husk dolls and woven amulets. In this study, corn was cultivated hydroponically, and the root fibres were employed for further investigation^30–32^.

### Surface modification using chemical treatments

The use of reagent functional molecules that can react with naturally occurring fibres that contain hydroxyl groups and the effective removal of non-cellulosic contaminants from the fibres are the main tenets of chemical treatment procedures^3,11,33^. Due to their hydrophobic nature, polymer matrices prevent full adherence and compatibility with hydrophilic natural fibres. One strategy for enhancing the interfacial qualities of fibres is to change the fibre surface by treatments that promote the chemical coupling of the fibre material to the matrix by displaying more reactive groups on the fibre surface^15,34–36^.

The modification is anticipated to diminish the fibre's tendency to absorb water by removing the hemicellulose, lignin, pectin, and wax that coats the fibre’s surface. This also serves to increase the physical and chemical coupling between the fibre and the polymer matrix by enhancing the alignment of the fibres, hence boosting fibre strength, and by slightly roughening the surface of the fibre to create micropores, which serve as the sites for interlocking. Furthermore, chemical treatment can lessen the creation of voids and improve the mechanical properties of the fibres^37–42^.

In order to create composites with improved properties, a variety of chemical treatment techniques have proven to be efficient. The types of chemicals employed in the treatment process have a significant impact on how well the composites turn out. Alkali treatment, permanganate treatment, and silane treatment are some of the most frequently used chemical treatment processes. Sodium hydroxide treatment has the lowest efficiency of any of the chemicals that develop a greater capacity to absorb water. The treatments with acrylic acid, silane, and potassium permanganate are the most effective at improving the water absorption ability of natural fibre-reinforced composites. In general, a natural fibre's interfacial adhesion to an epoxy matrix is improved by alkali and KMnO_4_ treatments^2,10,43–45^.

#### Alkali pre-treatment on natural fibres

Alkaline treatment, also known as mercerization, is one of the most often utilised chemical procedures for treating natural fibre to be used as a reinforcing material in thermoplastics and thermosets^46^. In the network structure between the chains of the cellular molecules, hydrogen bonding is lost, and new reactive bonds are formed as a result of alkalization^33^. The fibres' moisture resistance is enhanced, and there is also greater effective fibre surface area available for robust adhesion to the matrix^3^. Amorphous cellulose production rises, whereas the content of crystalline cellulose declines^28,34,47^. The alkali treatment shrinks the fibre's diameter, increasing the aspect ratio and causing the surface to become rougher; this increases the adherence of the fibre to the matrix. In addition, the alkali treatment expands the range of possible reactive sites, thereby improving fibre wetting. The hydroxyl group in aqueous sodium hydroxide is converted to alkoxide by ionisation^1,4,5,48–50^.

By removing the oils that cover the outside of the fibre's cell wall as well as other natural and artificial contaminants, alkaline treatment of natural fibre increases its tensile strength. By washing the surface, the alkaline reagent is utilised to alter the cellulose in the plant fibres' structure^6,22,38^. The composite fibre bundle is broken up into smaller fibres as a result of the process known as fibrillation, which follows mercerization. Thus, the extraction of lignin and hemicellulose components as well as the degree of polymerization are all strongly impacted by alkaline processing^36,45,51^.

There is some capillary action or raised surface tension when the mercerized fibre comes into contact with a moist matrix. The improved mechanical strength that results from mercerized fibres is mostly caused by a mechanical interaction between the rough fibre surface and the matrix^44,52^. Increase in the fibre's ductility and compression strength are observed after alkali treatment. The alkali-treated composite is superior to the untreated composite in terms of interfacial shear strength. Numerous research studies have found that mercerizing natural fibres increases the mechanical, thermal, and dielectric properties of natural fibre composites. The mercerized fibres pull in more resin, giving the composite a higher density^11,13,53,54^.

According to previous studies, a higher crystallinity index at lower alkali concentrations is favoured because it enhances the connection between the fibre and the matrix. The significant factors ultimately affecting fibre quality are the NaOH content, soaking and drying temperatures, and treatment time^5,55^. When natural fibre is thoroughly delignified using greater alkali concentrations, this can result in a weaker or damaged fibre. Beyond a specific optimal NaOH concentration, the composite's properties significantly decline^56,57^.

#### Potassium permanganate treatment on natural fibres

The chemical compound permanganate contains the permanganate group MnO^4^. The initial graft copolymerization and the generation of the cellulose radical in the permanganate treatment are both carried out by the extremely reactive Mn^3+^ ions through the synthesis of MnO^3−^ ions.^11,38,58^. In order to generate cellulose manganate, hydroxyl groups in cellulose combine with extremely reactive permanganate ions. During the procedure, a coupling reaction occurs^49^. The chemical interaction between the fibre and matrix is improved by this treatment, enabling superior matrix fibre adherence. This process lowers the fibre's capacity to absorb water^22,28^. The OH groups of the lignin constituents react with permanganate ions, freeing these groups from the fibre cell wall. Consequently, the fibre surface becomes rougher^33,53^. All such processes aim to clean the fibre from impurities, waxes, and moisture and increase its hydrophobicity^1,52,59,60^.

Permanganate treatment is a less popular but nevertheless beneficial treatment. Potassium permanganate (KMnO_4_) is employed in the majority of permanganate treatments^46^. The fibres are submerged in a potassium permanganate solution that has been dissolved in acetone or distilled water^57,61^. Natural fibre modification using KMnO_4_ in water solution, as compared to modification in acetone, is better for industrial applications, kinder to the environment, and does not require dangerous organic solvents^35^. In general, the permanganate treatment comes after the alkaline treatment^48^. One of the most efficient techniques used to enhance the binding at the fibre-polymer interface is the permanganate treatment that leads to changes in the macromolecular and crystallographic structure of the fibre composites as well as improved tensile characteristics, superior flexural strength, increased stiffness, and other attributes^4,6,15,40,42,51,56^. Rearrangement of microfibrils is facilitated by the delignification process that occurs during permanganate action; this improves crystallinity^29,44^.

The fibre properties will initially improve with increasing permanganate concentration before abruptly deteriorating once a particular threshold is achieved, suggesting the degradation of cellulosic fibres that will result in polar groups between the fibres and the matrix. As such, the fibre qualities depend strongly on the treatment's concentration and the duration of exposure^45,47,50,52,62^. According to some studies, treating natural fibre with KMnO_4_ can significantly lessen the amount of water that it can absorb. However, the hydrophilic nature of the aldehyde and carboxyl groups produced during KMnO_4_ treatment makes it difficult to hinder water absorption^35^. Additionally, permanganate-treated oil-palm fibre composites' tensile strength was reported to have fallen by 16%. In oil palm fibre reinforced composites, although the treatment aimed to increase interface interlocking using radicals, the resulting mechanical properties showed no improvement^55^.

Zea mays (Zm) root fibres were chemically pre-treated with alkali solution and then with permanganate solution in the present work. They were then tested for the changes in their qualities related to being better reinforcement materials for polymer composite-based products.

## Methods

### Growth and separation of *Zea mays* root fibres

The process of growth of corn plants by the hydroponic method used in this study is as follows:

- The maize seeds were cleaned, and any residual dirt was removed.
- The seeds were submerged in water for not more than 48 h.
- The water was drained. Then, the seeds were placed in a wet thick towel and kept wrapped for 48 h.
- The seeds were spread out in a tray (without overlapping), and then covered with a thin cloth as the leaves began to sprout.
- Every two hours, the towel was opened and the seedlings were watered.
- Hydroponically grown maize stems typically begin to turn red at six days of age.
- After 8–10 days, the seedlings were harvested. The roots were separated from the shoots. The grass can be used as fodder for animals.

### Surface modification using Permanganate treatment

**Figure 1:**
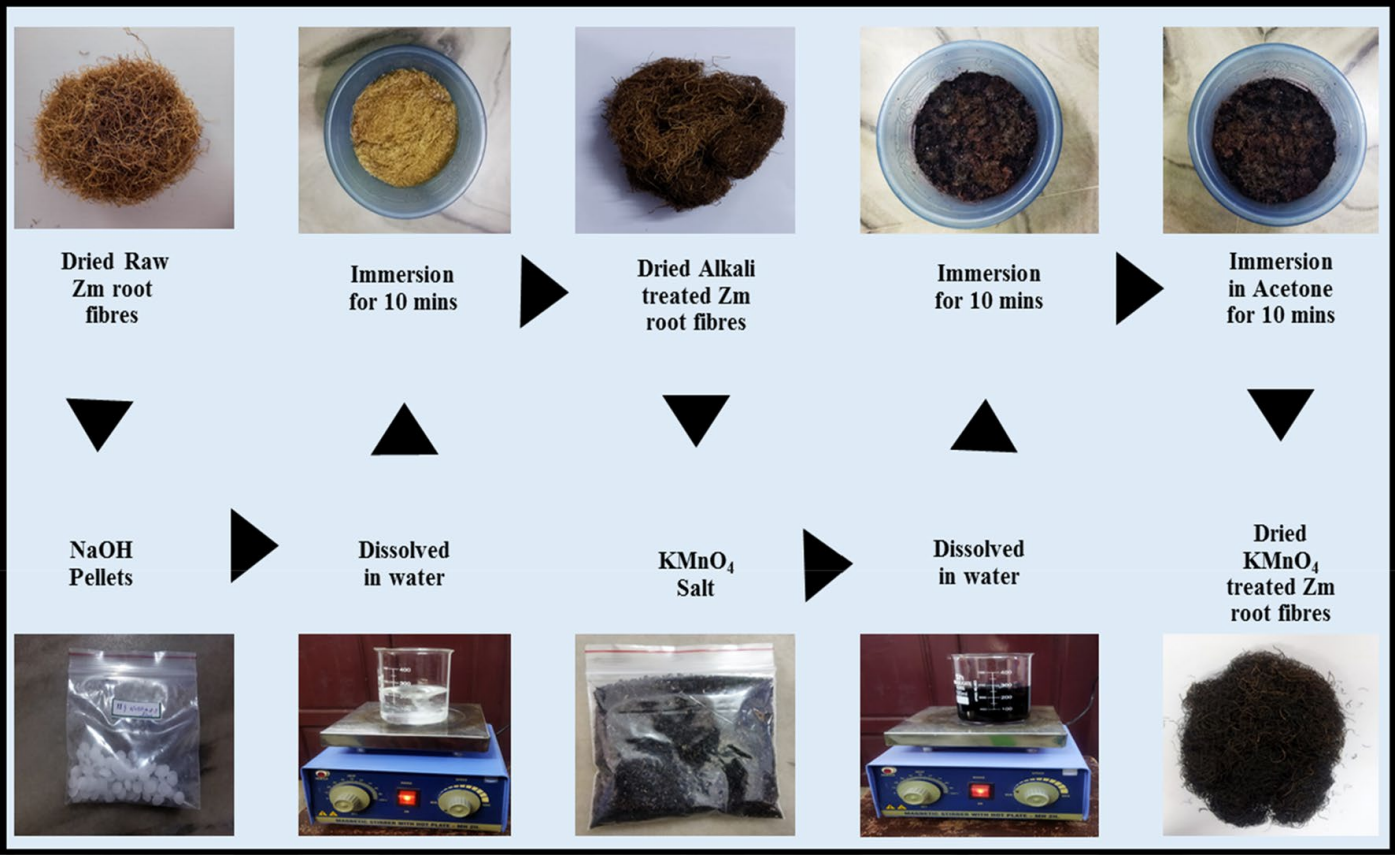
Permanganate Treatment of Zm root fibres.

The process of surface modification of root fibres are as follows:

- Dried raw Zm root fibres were obtained.
- The root fibres were pre-treated with sodium hydroxide, and a 0.1 M alkali solution was prepared. The fibres were submerged in the alkaline solution for around 10 min.
- The fibres were then dried at room temperature for one week.
- After they were completely dried, the alkali pre-treated fibres were soaked in a 0.1 M solution of potassium permanganate for 10 min.
- Immediately after the permanganate treatment, the fibres were immersed in acetone for about 10 min.
- During the following week, the fibres were dried at room temperature. The dried permanganate-treated Zm root fibres were sent out for various characterisation studies. Figure 1 shows the chemical treatment procedure of Zm root fibres.

### Characterisation studies

**Figure 2:**
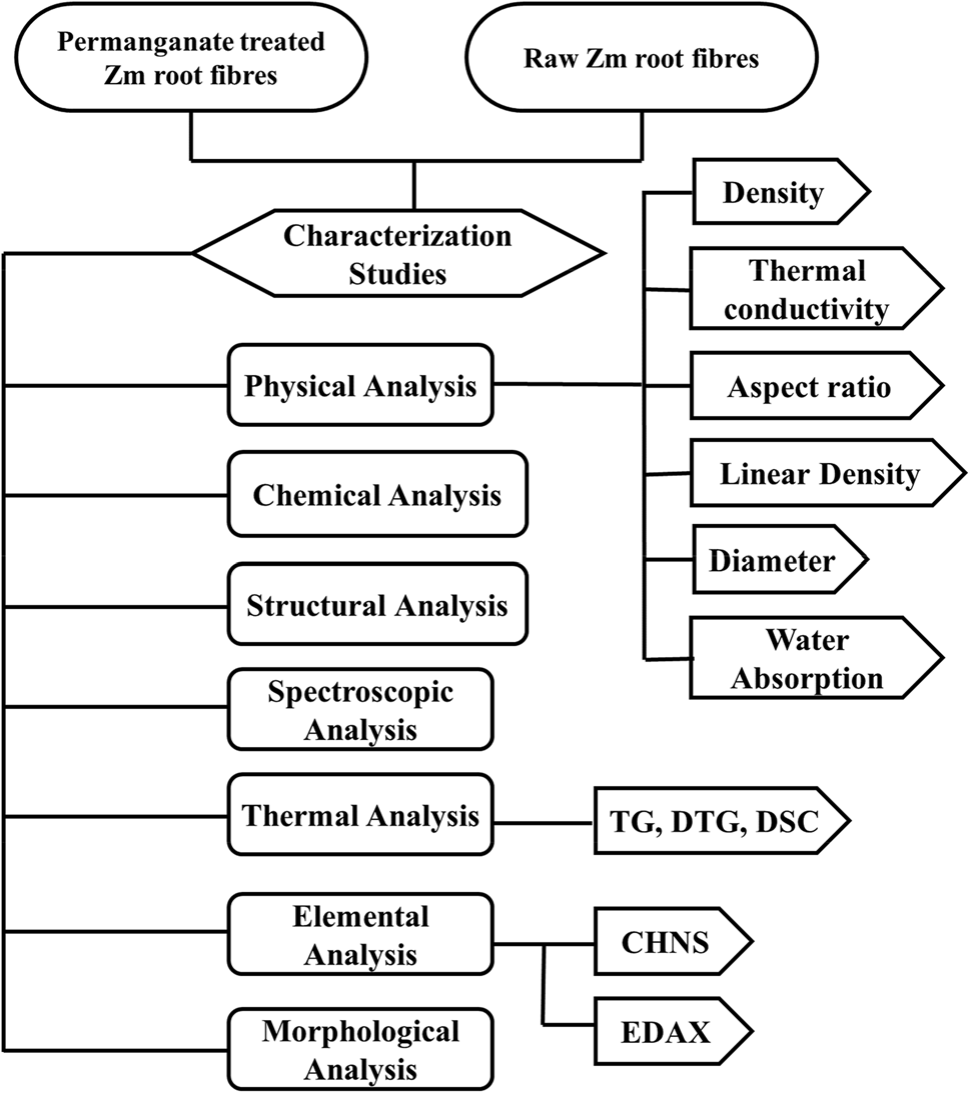
Characterisation studies for the analysis of Zm root fibres.

Characterisation of the samples obtained is mandatory to discern their properties and to observe the changes in their qualities due to chemical treatment. Figure 2 sketches the characterisation techniques used in the study of Zm root fibres.

#### Physical analysis

The natural fibres were subjected to the analysis of their physical traits. The fibres’ traits were carefully examined before being employed in polymer composites, as they have a considerable influence on the final quality of the composite material. Here, the physical characteristics of Zm fibres including their density, thermal conductivity, aspect ratio, linear density, diameter, and water absorption behaviour were studied. Chemical processing will alter the fibres' physical characteristics. These modifications underwent thorough analysis and tabulation.

##### Density

Pores and voids are typically present in natural fibres. As a result, their porous nature contributes to the difficulty in estimating a meaningful density value. The fibres were nevertheless measured for density in accordance with the ASTM D578-89 standard^15^. Toluene was utilised as the immersion liquid when employing the liquid pycnometer method to determine density. The formula for density is

1 $${\uprho }_{{\text{zmf}}} =\frac{\text{(m}{2}-{\text{m}}{1}\text{) }}{\text{(m}{3}-{\text{m}}{1}\text{)} \, - \, \text{(m}{4}-{\text{m}}{2}\text{) }}{\uprho }_{{\text{t}}},$$

where the Pycnometer mass (m_1_) is the weight of an empty dry pycnometer; m_2_ is the weight of the pycnometer and fibre; m_3_ is the weight of the pycnometer and toluene, and m_4_ is the weight of the pycnometer plus toluene and fibre. ρ_t_ is the density of toluene, and ρ_zmf_ is the density of Zm fibres^16^.

Three assessments were made for the density tests, and the average was taken as the value. Before each measurement, it is necessary to ensure that no air bubbles were formed when the toluene was added to the pycnometer^12^.

##### Thermal conductivity from Lee’s Disc

**Figure 3:**
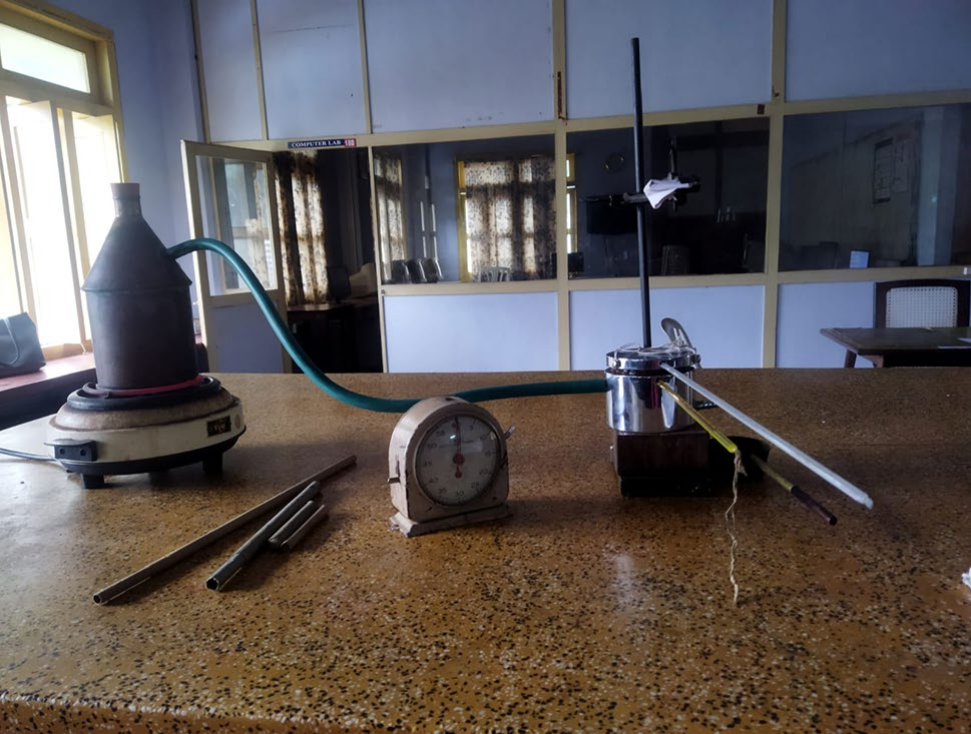
Lee’s Disc Experimental set-up.

The most important consideration when evaluating a material's thermal response is its coefficient of thermal conductivity (K). Stronger heat insulation is indicated by a smaller K value of the material. Greater porosity in a material results in a lower heat conductivity coefficient of that material. The material's components, porosity, pore size, features and water ratio all affect the material's coefficient of heat conductivity. The thermal conductivity of natural fibres typically ranges from 0.0341W/mK to 0.0599 W/mK^63^. Lee's disc test can be used to determine the thermal conductivity of natural fibres. The experimental setup is shown in Fig. 3. Both Zm root fibre samples were moulded into a shape of disc manually with a radius of 5 cm. The experiment was repeated three times with three specimens each and the average value was considered. The following equation is used to obtain the thermal conductivity coefficient.

2 $$\mathrm{K }=\frac{\text{mc(dT/dt) x}}{{\text{p}}{\text{R}}\text{2 }\text{(T}{2}-{\text{T}}{1}\text{)}}. \frac{\text{R+2h}}{\text{2(R+h)}} {\text{W}}/{\text{mK}},$$

where K is the sample's coefficient of thermal conductivity; m is the metal disc's mass; c is the heat capacity, and dT/dt is the rate at which the metal disc cools. (T_2_—T_1_) is the temperature difference over the thickness of the sample, and x is its thickness; R is the sample’s radius, and h is the metal disc's thickness^30^.

##### Aspect ratio

By dividing the fibre's length by either its diameter or thickness (both measured in the same units), the aspect ratio can be computed. Both the diameter and the length of the fibres are measured using a compound microscope of magnification (40–1000 ×) and a measuring scale. A higher ratio means that the fibre is more flexible. It has been found that the aspect ratio, fibre content and fibre dispersion should be in ascending order to increase the quality of composite materials. The average diameter and length of 25 Zm root fibres were measured. The length of the fibres were within 10–15 cm. All measurements were made at room temperature. The following equation is used to calculate the aspect ratio of plant fibres.

3 $$ {\text{Aspect ratio }} = {\text{ L}}/{\text{D}}, $$

where L stands for the average fibre length, and D is the average fibre diameter^29^.

##### Linear density

The measurement of the quantity of any characteristic value per unit of length is called linear density. It is possible to learn more about a fibre's quality by reference to the ASTM D1577 standard's guidelines. For this procedure, at least ten to twenty Zm root fibres must be examined for consistency^64^. Average length was measured using a measuring scale and average mass was determined using a digital balance. The linear density of the fibres can be calculated using the formula below:

4 $$ {\text{Linear density }} = {\text{ M}}/{\text{L g}}/{\text{km}}, $$

where L is the fibres' average length, and M is the average fibre mass^16^.

##### Diameter

Natural fibre diameter is generally measured under a compound microscope. An air wedge (± 0.001 mm) is used as the measurement technique. Most natural fibres have an uneven shape and varying thickness, making it difficult to gauge their diameter with precision. The fibre diameter is found by measuring three to four times along the length (for consistency, 10–15 fibres are normally analysed)^64^.

##### Water absorption behaviour

A crucial factor in replacing synthetic fibres in the development of natural fibre polymer composites is the degree of adhesion between the hydrophobic polymer and the hydrophilic natural fibre. Numerous natural fibre constituents contain hydroxyl groups, resulting in excessive water absorption, poor wettability, and weak interfacial interaction involving the polymer matrix and cellulosic fibres. Therefore, for producing composites it is essential to increase the hydrophobicity of natural fibres with acceptable and improved mechanical properties. This can be done by giving the fibre surface an appropriate chemical treatment. One of the crucial characteristics for determining the hydrophobic character of the fibre material is its ability to absorb water^54^.

To measure water absorption, samples were immersed for five minutes at a time in separate containers filled with 1000 ml of water for 30 min at room temperature. Each sample for this procedure weighed eight grams. Samples were removed from the container at predetermined intervals (every 5 min), and the fibre samples were held between two sheets of filter paper. The moisture on the fibre surface is consequently absorbed by the filter paper. The next step involves using a precision digital balance to weigh the sample to calculate the amount of water absorption^65^.

5 $$\mathrm{Water \,Absorption \% }= \frac{Weight\, after\, immersion-Weight\, before\, immersion}{Weight\, before \,immersion}* 100\mathrm{\%}$$

#### Chemical analysis

Natural fibres' chemical makeup has a significant impact on their physical characteristics and thus will influence the characteristics of composites consisting of natural fibres. Cellulose is a crucial component for giving natural fibres the appropriate rigidity and strength. The structure of cellulose and its crystallinity affects the reinforcement efficiency of natural fibres. Therefore, the fibre strength increases with the cellulose content. Oxidising agents cannot harm cellulose. The moisture absorption in the fibres is due to the hemicellulose. Therefore, the quantity of hemicellulose must be reduced by utilising different surface treatments. Hemicellulose is hygroscopic and only partially soluble in water because it is tightly attached to the cellulose fibrils, most likely via hydrogen bonds. Hemicelluloses are easily degraded by acids and dissolve in alkali^53,66^.

The lignin content of fibres determines their structural behaviour, morphological characteristics, and behaviour regarding fire resistance. It has been demonstrated that natural fibres' capacity to stretch is increased by a high lignin concentration. Lignin is amorphous, extremely complex, and hydrophobic. Lignin is easily oxidised, soluble in hot alkali, and not hydrolysed by acids. Another element, pectin, is responsible for the cellulose fibres' attachment to other fibre elements. Lignin and pectin must be eliminated to provide efficient composite reinforcement since they are weaker amorphous polymers than cellulose. Plants gain their flexibility from pectin. Cellulose, hemicellulose, lignin, and pectin are the main components of natural fibres. Since other polymers are present in negligible percentages, they barely affect the fibre structure and characteristics^9,29,36,41,66^.

To produce ash, a certain number of fibres are burned in platinum crucibles over open flames or in a muffle furnace. The residue that is left over is then weighed. The amount of residue determines the ash content. The amount of ash in the fibres has no impact on the mechanical characteristics of composites. Ash concentration in natural fibres is lowest, as a result, their impact on fibres is less. The final component of fibres is wax, which is made up of multiple alcohols^67^.

The amounts of cellulose and other amorphous substances present in Zm root fibres was determined by chemical analysis. Cellulose content was measured using the method SITRA/TC/FCC/01; hemicellulose was measured using the method SITRA/TC/FCC/05; the lignin content was measured using the method: SITRA/TC/FCC/02, and the pectin was measured using the method SITRA/TC/FCC/04. The ash content was measured using the method IS 199, and the same method was again utilised for the moisture content. The wax content was measured using the method SITRA/TC/GT/09. Finally, the density was measured using the method SITRA/TC/FCC/03. All these methods are specific techniques followed to determine the chemical constituents of any natural fibre by SITRA, Coimbatore, Tamil Nadu, India, where the samples were sent for chemical analysis^23,68^.

#### Structural analysis using p-XRD

The method for determining whether a material is crystalline or amorphous is called Powder X-ray Diffraction (p-XRD). This method is employed to assess the degree of crystallinity within fibres. Crystallinity is a metric used to describe the way cellulose crystals in fibres are oriented in relation to the fibre axis. The elimination of waxy substances, lignin, pectin, cementing substances, and hemicelluloses may increase crystallinity by making the interfibrillar areas less hard and dense and improving the arrangement of the cellulose chains. Since many fibres are semi-crystalline by nature, the changes seen in the fibre samples can be precisely defined and analysed using the p-XRD method^54^.

A Bruker model D8 Advance A25 X-ray diffractometer was used to analyse powdered Zm fibre samples produced by slicing the Zm fibres into thin, powder-like strips. The XRD investigation was conducted at an operating temperature range of 3° to 70 °C, with a scanning rate of two scans per minute. The formula below was used to calculate the samples' percentage of crystallinity.

6 $$\mathrm{\% \,Crystallinity }= \frac{{I}_{200}}{{I}_{200+{I}_{am}}}\times 100$$

where I_200_ and I_am_ are the crystalline and amorphous intensities at 2θ scale close to 22° and 16°, respectively. The size of the crystallite (CS) is determined using Scherrer's formula:

7 $$\mathrm{Crystallite size }=\frac{K\lambda }{\beta {\text{cos}}\theta },$$

where K = 0.9; **λ** = 1.54060 × 10^–10^ m; β = p/180 × FWHM; θ = Bragg’s angle^23,30,69^.

#### Spectroscopic analysis using FTIR

Fourier Transform Infrared analysis (FTIR) is a quick, accurate analytical technique used to identify the components of organic materials on a qualitative level. The chemical structure of the Zm root fibres was examined both before and after the treatment to identify the functional groups and any surface alterations. This procedure increases the level of confidence in material confirmation and identification^34,58,59,70^.

Perkin Elmer Spectrometer in interferogram mode with a resolution of 2 cm^−1^ was used for the analysis. Zm root fibres were combined with Potassium bromide (KBr) solution, and the resulting mixture was compressed using a hydraulic press to yield pellets. The Zm fibre pellets were subjected to IR radiation with a wavenumber range of 400 cm^-1^ to 4000 cm^−1^, and 32 scans were taken to record the fibres' spectra. The graph of transmittance versus wavenumber is presented in the form of an IR radiation spectrum^4,66^.

#### Thermal analysis

Before using a natural fibre as reinforcement in a polymer matrix, its thermal stability must be examined in order to fully comprehend the curing and extrusion temperatures of thermoset and thermoplastic matrix composites, respectively. To study the thermal stability of Zm fibres, Thermogravimetry (TG) and Differential Thermogravimetry (DTG) techniques can be utilised. The durability of composite materials using plant fibre reinforcement can be increased if the natural fibres possess better thermal properties^29,71,72^.

A Perkin Elmer Diamond TG/DTA analyser was used to investigate the thermal behaviour of powdered Zm samples. Alumina crucibles with pinholes were used to hold approximately 10 mg of samples, which were then heated to a maximum temperature of about 750 °C at a constant rate of 10 °C/min and a flow rate of 100 ml/min. Three specimens were measured for each tested sample. The resulting graph for raw and modified Zm fibres is displayed to show the mass loss of the fibre as a function of heating temperature^4,11,40^.

##### Thermal activation energy

Utilising TG analysis, which is also used to make prospective energy recovery forecasts once the compounds approach the end of their useful lives, one may determine the kinetic properties of natural fibres and their bio-composites^73^. A higher activation energy indicates that more energy is needed to break the bonds, i.e. an improved fibre-matrix adhesion of bio-composites. The thermal stability of fibres increases with higher activation energy. Another aspect of thermal activation energy is the bare minimum of energy needed to begin the breakdown of fibres^74,75^.

The method suggested by Coats and Redfern is most frequently employed in the kinetic investigation of thermal degradation of biomass. The slope and intercept of the regression line can be used when calculating the activation energy (E_a_). Thus, using the Coats-Redfern approximation, the kinetic activation energies of unmodified and modified Zm fibres are estimated from TG data^29^.

8 $${\text{log}}\left[\frac{-{\text{log}}\left( 1- \alpha \right)}{{T}^{2}}\right]={\text{log}}\frac{AR}{\beta {E}_{a}}\left[1-\frac{2RT}{{E}_{a}}\right]-\frac{{E}_{a}}{2.303 RT}$$

where T is the absolute temperature; *β* is the linear heating rate; A is the frequency factor; *E*_*a*_ is the activation energy; R is the gas constant, and α is the fraction of decomposed sample at time t. The value of α is calculated as α = $\frac{{W}_{0}-{W}_{t}}{{W}_{o}-{W}_{f}}$, where W_o_ is the initial sample weight (before starting the decomposition reaction); W_t_ is the sample weight at any given temperature, and W_f_ is the final sample weight after completion of the reaction. A linear fitting of log $\left[\frac{-{\text{log}}\left( 1- \alpha \right)}{{T}^{2}}\right]$ versus $\frac{1000}{T}$ is done to calculate the activation energy^30^.

##### DSC

The thermal transitions of unmodified and chemically treated Zm root fibres were examined using a Mettler Toledo DSC analyser (model DSC 822e). Three grams of fibre samples in each condition are placed in an aluminium pan, and the pan is heated to 750 °C. Throughout the analysis, a heating rate of 10 °C/min is maintained within a temperature accuracy of ± 0.2 °C^76^.

#### Elemental analysis

The process used to learn more about an unknown substance's elemental composition is called elemental analysis^77^. Here elemental composition of both Zm fibre samples was learnt using two methods: CHNS and EDS.

##### CHNS (Carbon Hydrogen Nitrogen Sulfur Analysis)

Permanganate-treated and untreated Zea mays root fibres (Zm) were analysed for their elemental composition using an automated Elementar Vario EL III model CHNS/O Elemental Analyzer. In order to ascertain the concentrations of carbon, hydrogen, nitrogen, and sulphur in a sample, an elemental analysis technique known as CHNS analysis was performed. The sample was burned in an atmosphere with sufficient oxygen. While burning, the elemental analyser emits homogeneous gases of C, H, N, and S. The detection of the gases can be accomplished in a number of ways, such as separation followed by quantitative analysis using thermal conductivity detection, partial separation (referred to as "frontal chromatography") followed by thermal conductivity detection, or a series of independent infrared and thermal conductivity cells for compound-specific detection^23^. Three specimens were measured for each tested sample and the average value was considered.

##### EDS

EDS is used for elemental detection. The JEOL 6390LV model with a range of 0.5 kV to 30 kV, a resolution of 4 nm, and a 300,000 magnification was used for the sample analysis. Each sample was analysed three times, and the average was used as the final result.

#### Morphological analysis using SEM

The influence of permanganate treatment on the surface of the Zm root fibres was evaluated using a scanning electron microscope model Joel 6390 LV with an accelerating voltage range of 0.5 to 30 kV. Before SEM analysis, all specimens were double-sided coated with an electrically conductive carbon-adhesive tab. To improve image resolution, a gold layer of 10 nm was sputtered onto the fibre and mounted on holders made of aluminium. Different surface locations and magnification settings were used to assess the surface structure of the fibres. The diameter of Zm fibres was calculated from the acquired SEM images using ImageJ software^3,4,11,29,70^.

### Guidelines and regulations

All testing was performed in accordance with the relevant ISO standards. The methods and procedures used were compliant with the guidelines and regulations outlined in the ISO standard to ensure accurate and reliable results.

### Specimen collection

No specimens were collected from private or public land. All cultivation and experimentation were conducted within the author's premises without requiring external permissions.

## Results and discussion

### Physical analysis

In general, NaOH and KMnO_4_ treatment of fibres will stop moisture from entering the core of the composites, allowing them to withstand loads and postpone the onset of fracture. In this study, the physical features of Zm root fibres after permanganate treatment were investigated.

#### Density

Reduced density is the main factor in the commercial viability of natural fibre-based products over synthetic materials^12^. Surface alterations have a small impact on fibre density. This is because the reactions mostly impact the surface of the fibres and do not affect their core structure. ’The cellular structure of Zm fibres is destroyed after permanganate treatment, and this lowers the void content^71^. By eliminating less dense non-cellulosic elements such as lignin and hemicelluloses, permanganate treatment enhances the density of fibres. The change in density is brought about by chemical modifications that reduce the crystal defect and increases the density of the cell walls of the fibres, thereby increasing the density of the fibres^41,54^. Table 1 below specifies the physical parameters of raw and permanganate treated Zm fibres.

**Table 1:** Physical parameters of Zm root fibres.

Sample	Density using a pycnometer (g/cm^3^)	Coefficient of thermal conductivity (W/mK)	Aspect ratio	Linear density (tex)	Diameter using a microscope (µm)
KMnO_**4**_-treated Zm root fibres	0.9241	0.04189	392.1568	707.26	248
Raw Zm root fibres	0.7694	0.02990	446.1766	1509	345

The densities of *Acacia pennata* fibres (1.090 g/cm^3^) and *Acacia leucophloea* (1.385 g/cm^3^) and coir fibres (1.200 g/cm^3^) are similar to permanganate-treated Zm root fibres. While *Acacia planifrons* (0.660 g/cm^3^), *Prosopis juliflora* (0.580 g/cm^3^) and alkali-treated Zm root fibres (0.557 g/cm^3^) have density values lower than permanganate-treated Zm fibres. Jute fibres (1.460 g/cm^3^), flax fibres (1.500 g/cm^3^), ramie fibres (1.500 g/cm^3^), and cotton fibres (1.600 g/cm^3^) have greater density values^30,70^.

#### Thermal conductivity from Lee’s Disc

**Figure 4:**
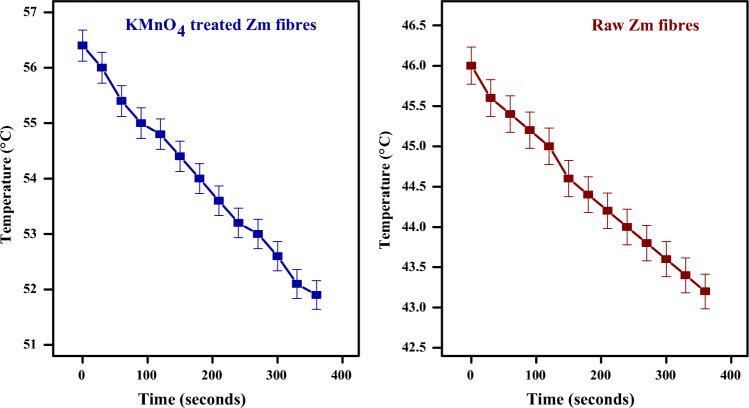
Thermal conductivity graph of Zm root fibres.

One of the most crucial factors in the development of highly energy-efficient products is the structural materials' ability to provide thermal insulation. Due to their strong thermal insulation capacity and low thermal conductivity, fibres can be utilised as insulators at construction sites. The physical appearance of Zm fibres is a further crucial factor. Since air conducts heat poorly, any material that contains air will likely have lower heat conductivity than is common. The physical structure of Zm fibres is particularly porous, with air filling approximately one-third of the volume as a result of the numerous cavities that make up the fibre structure. As a result, the fibre has a low ability to conduct heat^78^. Figure 4 shows the temperature vs time plot to determine the thermal conductivity of Zm root fibres.

Conductivity (K) values for untreated and KMnO_4_-treated fibres were 0.029 W/mK and 0.041 W/mK, respectively (Table 1). The permanganate treatment of Zm fibres resulted in a slight increase in thermal conductivity. This was lower than the heat conductivity of several other natural fibres. Areca husk fibres (0.021 W/mK), flax (0.038–0.075 W/mK), coconut fibres (0.058 W/mK), sugarcane fibres (0.0469 W/mK), cotton stalk fibre boards (0.0585–0.0815 W/mK) and hemp fibres (0.040–0.060 W/mK) are a few of the well-known natural fibres with comparable thermal conductivity values. Such fibres may be utilised in ceiling applications. When included in composites, lower K values provide the possibility of using Zm fibres for insulation purposes^29,30^.

#### Aspect ratio

The aspect ratio (L/D) of an object is the ratio between its longest and shortest dimensions. Structures with a high aspect ratio have features that are quite tall while not being very wide. Long, thin fibres often exhibit better qualities, but they are more expensive to create and may be more challenging to disseminate evenly throughout the composite. Although composites made with small aspect ratio fibres often have greater compressive qualities, they typically have lower damage resistance. Table 1 lists the aspect ratios of permanganate-treated and unprocessed Zm fibres. Since the aspect ratio of the raw Zm fibre was altered after treatment, the effect of potassium permanganate treatment on Zm roots are apparent.

#### Linear density

Linear density is the term used to denote the mass or weight of a fibre per unit length. The qualities of the finished product, including strength, elasticity, and durability, can be greatly influenced by linear density, making it a crucial metric. A fibre with a higher linear density is thicker, heavier, potentially stronger, and more durable. However, a material that has a higher linear density may also be stiffer and less flexible. In contrast, a fibre with a lower linear density is thinner, lighter, and may be softer and more flexible, but it is also less strong. Fibre tensile characteristics improve with increasing linear density. The linear densities of both samples were analysed and are listed in Table 1. The results showed that there was a decline in the linear density of the sample following treatment.

#### Diameter

Table 1 shows the difference in diameter brought on by chemical treatment. The loss of the outer layer caused the fibre's diameter to decrease. The fibre modulus increases as the fibre diameter decreases. The diameter of the fibre also changes with the layer of the plant. Zm fibres were treated with permanganate to lower their diameter, and this increased their compactivity with the matrix. Zm fibres had a diameter equivalent to that of *Acacia pennata* fibres (299.39 μm). Zm root fibres treated with potassium permanganate had a smaller diameter than many other natural fibres^41,70^.

#### Water absorption behaviour

**Figure 5:**
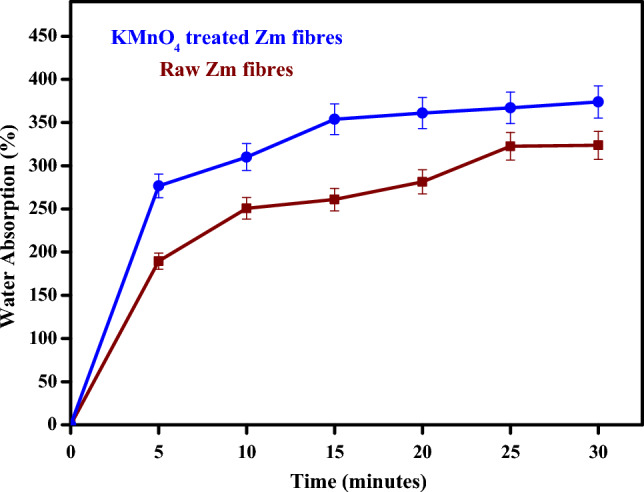
Moisture absorption graph of Zm root fibres.

Figure 5 displays the moisture gain of raw Zm root fibres and the fibres after treatment with KMnO_4_. In comparison to untreated fibres, treatment with permanganate solution resulted in increased moisture gain^39^.

The raw Zm root fibre absorbed a lesser percentage of water than the permanganate-treated Zm fibres at (Table 2). The raw fibre is encased with natural oil and wax and thus may absorb less water; this is the most likely explanation for the decrease in absorption percentage. Because they contain less oil and wax than raw fibres and because hydrophobic lignin has been removed from their fibre structure, the permanganate-treated fibres exhibited better water absorption. This may also be caused by the presence of numerous pores created due to treatment on the fibre's surface and interior, as the pores can trap water molecules^66^. After being treated with permanganate, the cellulose concentration also decreased, thereby increasing the fibre's hydrophilicity. The moisture absorption percentage of raw Zm fibres (189.60–323.62%) was comparable to alkali-treated PALF fibres (175–300.5%). Likewise, the absorption percentage of permanganate-treated Zm fibres (276.66–373.81%) was comparable to alkali-treated banana fibres (248.5–480%). Raw areca fibres (698–851%) had a high absorption percentage, while raw cotton fibres (32.5–50.5%) had a low absorption percentage among various natural fibres^65^.

**Table 2:** Moisture Absorption behaviour of Zm root fibres.

Sample	Water absorption (%)					
	5 min	10 min	15 min	20 min	25 min	30 min
KMnO_**4**_-treated Zm root fibres	276.66 ± 13.83	310.08 ± 15.50	353.80 ± 17.69	361.08 ± 18.05	367.03 ± 18.35	373.81 ± 18.69
Raw Zm root fibres	189.60 ± 9.48	250.71 ± 12.53	260.91 ± 13.04	281.28 ± 14.06	322.52 ± 16.12	323.62 ± 16.18

One issue that needs to be clarified is that the outcomes of the water absorption tests are not always related to the fibres' typical moisture content. When materials are exposed to the surrounding environment, they gradually take in moisture from the air. In this investigation the amount of water absorbed was assessed after submerging the fibres in water for a predetermined amount of time. This absorption occurred due to direct contact with the water.

Finally, the results supported the proposal that permanganate-treated fibres have a higher water absorption percentage and can be employed in the technical textile industry due to their porous structure. Raw fibres can be utilised in situations where relatively low levels of water absorption are required since they absorb less water^65^.

### Chemical analysis

In general, the presence of lignin and hemicellulose is explained by the insufficient interfacial bonding of fibres in the polymer matrix. After permanganate treatment, the percentages of lignin and hemicellulose were decreased. Reduced hemicellulose, lignin, wax, and moisture contents in treated Zm root fibres indicated an increase in the tendency towards a more packed crystalline order and improved mechanical properties. Chemical treatment thus helped to remove impurities, wax, and moisture, and to expose the micropores of fibres such that the resin can build up on the fibre surface more rapidly than on untreated fibres. In this regard, surface alteration will result in increased bonding between the fibres and the matrix^40,79^. Table 3 below tabulates the chemical composition of Zm fibres.

**Table 3:** Chemical analysis of Zm root fibres.

Chemical composition	KMnO_4_ treated Zm root fibres (%)	Raw Zm root fibres (%)
Cellulose	55.5	58.74
Hemicellulose	20.4	29.53
Lignin	10.2	19.04
Pectin	5.6	4.69
Wax	0.56	1.37
Ash content (on dry basis)	16.3	3.47
Moisture content	11.7	11.86
Density (g/cc)	1.13	0.64

Modified Zm root fibres had a relatively high density, primarily due to the removal of contaminants using solvents, the lower wax content, and the filling of fibre pores with grafted molecules. The ash content of treated fibres was increased. Since the influence of ash content is the least in the properties of fibres, this could be averted^11,34,40^.

The high cellulose contents of raw and permanganate-treated Zm root fibres are advantageous. *Zea mays* has cellulose levels that are equivalent to those of other natural fibres such as *Acacia concinna* (59.43%), Sugarcane bagasse (55.2%), Abaca (56–63%), Rice straw (57%) and more substantial than those of Coir fibres (32–43%), Kenaf fibres (31–39%) and *Ficus* leaf fibres (38.10%)^70^.

### Powder X-ray diffraction analysis

**Figure 6:**
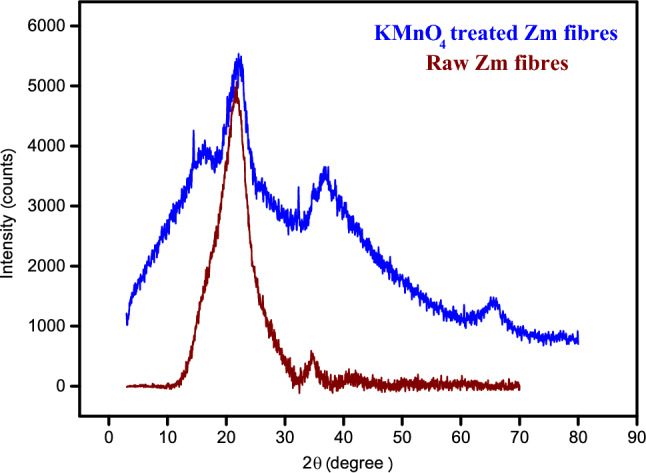
p-XRD pattern of Zm root fibres.

An X-ray diffraction investigation is carried out to determine the degree of crystallinity and the crystal structure of the chosen fibres^60^. Figure 6 shows the diffraction patterns of unprocessed and permanganate-treated Zm fibres.

The amorphous (110) and crystalline (200) components of the fibres are shown by the two prominent peaks at 16° and 22° that are especially well defined for natural fibres. The wide peak at 22° clearly shows that the permanganate solution permeated the fibre and removed the wax, hemicellulose, and lignin from the surface. The existence of cellulose is indicated by the crystalline peak at 2θ = 22°. Cellulose is a crucial structural element that gives plant fibres strength and hardness. Higher cellulose content, greater crystallinity, and larger crystallite size are thought to improve the thermal properties of fibre materials because these factors increase the stability of the fibre by preventing thermal expansion caused by intramolecular hydrogen bonding^2,29,35,40^.

The crystallinity percentage of permanganate-treated Zm fibres was 57.53%^4,61,80^. This was higher than many other natural fibres such as Karnataka sisal fibre (53%), *Grewia tilifolia* (41.7%), *Lygeum spartum* (46.19%), *Sansevieria ehrenbergii* (52.27%), *Acacia pennata* (46.52%), *Nelumbo nucifera* (48%) and *Furcraea foetida* (52.6%)^60^. The value was comparable to treated *Prosopis juliflora* (57.87%), treated Kapok fibre (58.42%) and sugarcane bagasse fibre (50%)^34^. Some natural fibres have even higher crystallinity, for example *Althaea officinalis* (68%), flax (70%) and jute (71%)^35^.

The crystallite size (CS) of the permanganate-treated Zm fibres was 2.2 nm (Table 4). The vacuum spaces in the unprocessed Zm fibres have been filled by the molecules of potassium permanganate. The size of the crystallite increased as a result. Table 4 presents the calculated values for the size of the crystallites and the crystallinity percentage of Zm root fibres^23^. The larger crystallites observed in treated fibres may potentially be connected to decrease in crystal distortion and defect^14^. By having a higher CS than raw root fibres, chemically treated Zm fibres demonstrate higher chemical reactivity^68^. The CS of Zm fibres was comparable to those of *Thespesia populnea* (3.57 nm), *Ferula communis* (1.60 nm), Cotton (5.5 nm), *Acacia pennata* (1.91 nm) and Flax (2.8 nm)^29,70^. However, since lignin is being removed along with cellulosic components during treatment, a drop in the degree of crystallinity was observed in Zm fibres, possibly as a result of the increased concentration of chemicals used^15^.

**Table 4:** Crystallographic information of Zm root fibres.

Sample	Percentage Crystallinity (%)	Crystallite size (nm)
KMnO_**4**_-treated Zm root fibres	57.53	2.2
Raw Zm root fibres	71.93	1.45

### Fourier transform infrared spectroscopy

Table 5 below shows the wavenumber and its relation to several functional groups associated with Zm root fibres. The allocations were based on the literature values for diverse natural fibres^41^.

**Table 5:** Vibrational band assignments of Zm root fibres.

Wavenumber (cm^−1^)		Vibrational band assignments
0.1 M KMnO_4_ treated Zm fibres	Untreated Zm fibres	
3436.65	3434.44	Stretching of cellulose by hydrogen-bonded O–H bonds
2923.18	2923.63	Cellulose C–H stretching
2853.16	2852.46	Hemicellulose stretching vibration with C–H symmetry
–	2053.87	Wax or material resembling wax
1639.71	1632.10	Acetyl group in hemicellulose shown by the carboxyl stretch of the C-O molecule
1452.99	–	CH_2_ bending of cellulose
1418.93	1408.97	C–H_2_ wagging
1383.75	1384.04	Asymmetric lignin COC stretching
1389.09	–	C–O groups of the aromatic ring in polysaccharides
–	1269.02	C–O stretching vibration in lignin due to acetyl group
1111.87	1109.01	C–O lignin ring
1060.97	1054.05	C–O stretching
1037.15	1036.97	CO group of cellulose
–	897.17	Lignin components
848.09	873.40	Monosaccharide ring and β-glycosidic linkage
780.44	778.32	Stretching of CO
–	617.00	Ring structured out-of-plane bending vibration
557.82	–	C–X stretching of organic halogen compounds
517.41	–	Bending of OH out of plane

**Figure 7:**
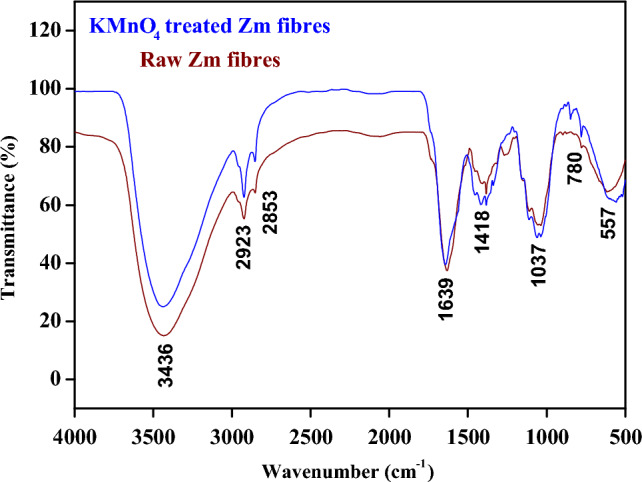
FTIR spectra of Zm root fibres.

Figure 7 shows the Fourier-transform infrared spectra of *Zea mays* root fibres. Both fibre samples had comparable peaks. However, the intensity of the peaks changed as a result of the treatment. There was a wide and strong peak within the range of 3430 cm^−1^ due to the hydrogen-bonded OH stretching of cellulose. This denotes the diminished hydrogen bonding with cellulose hydroxyl groups, resulting in less hydrophilicity of fibres. There was a slight shift in the peak position around 1630 cm^-1^ in the treated sample. The elimination of hemicellulose may have caused this shift, as this exposes the OH groups in the cellulose. The reinforcing fibre may be able to mechanically intertwine with the polymer matrix more firmly due to the greater degree of exposure^1^. In the treated fibres, vibrations brought on by amorphous groups essentially disappeared. The peaks at the wavenumber range 1410 cm^−1^ in both the samples indicated the change in the angle between the planes of atoms of CH_2_. The peaks around 780 cm^−1^ was due to C-H bonds that can bend above and below the plane of the molecule in the similar fashion. C-X stretch was shown by the peak at 557 cm^−1^ in the raw Zm root fibre sample which is generally intense because C-X bonds tend to have large dipole moments due to the electronegativity differences between carbon and the halogens. The absence of the minor peak at 897 cm^−1^ in the treated sample was due to permanganate activity causing the oxidation of lignin. Similarly, the peak showing the presence of wax at around 2050 cm^−1^ disappeared in the spectra of treated Zm fibres. Since many functional groups of amorphous materials such as impurities, lignin, hemicellulose, and waxes had been eliminated due to KMnO_4_ action, specific slight differences in peaks were detected for permanganate-treated Zm samples. The results supported the findings from the chemical examination of Zm root fibres^14,21,29,54,81^.

### Thermal analysis

The thermal stability of materials was evaluated using TGA. DTG curves showed the maximal rates of breakdown for weight loss of the compounds found in natural fibres. When choosing materials for specific end-use applications, TGA-DTG measurements offer useful information. Mass loss associated with Zm fibres were identified using Table 6 as provided below.

**Table 6:** Mass loss with temperature obtained from the TG curve.

Sample	Temperature during mass loss (°C)	Mass loss (%)	Residual char at 700 °C (%)
KMnO_4_-treated Zm root fibres	32–184	11.73	20.32
	184–331	32.41	
	331–511	34.81	
Raw Zm root fibres	28–120	10.07	0.076
	120–345	54.27	
	345–525	35.49	

Table 7 provides the peak temperatures, mass loss, and T corresponding to a 50% mass loss. TGA is one of the most popular methods for assessing the composition and structural sensitivity with thermal degradation of natural cellulosic fibres. Higher degradation temperature is generally caused by an enhanced crystalline structure. From the p-XRD analysis, the crystallinity percentage of raw Zm fibres were higher than that of treated fibres, as was the maximum degradation temperature^15,72^.

**Table 7:** T_max_ limit for Zm root fibres.

Sample	Total mass lost (%)			Max. Temperature Limit (°C)	T_(50%)_ (°C)
	First stage	Second stage	Third stage		
KMnO_4_-treated Zm root fibres	11.73	44.14	78.95	511	387
Raw Zm root fibres	10.07	64.34	99.83	525	309

**Figure 8:**
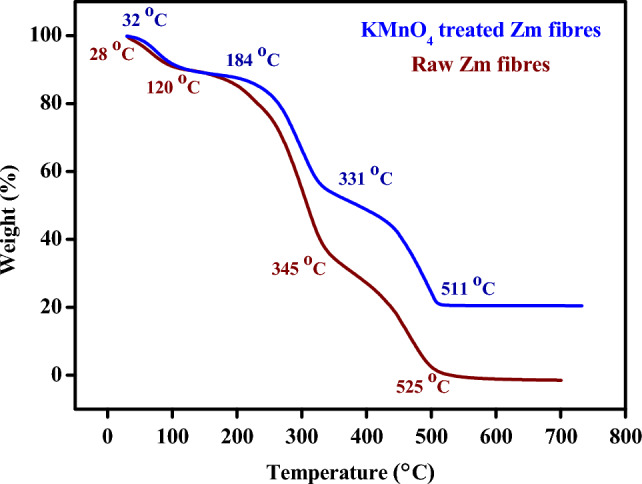
TG curve of Zm root fibres.

The fibres’ response to different temperatures was investigated using the TGA data. Both samples exhibited a three-step degradation (Fig. 8). An initial mass loss of 11.73% occurred between 32 and 184 °C as a result of the treatment, primarily due to moisture evaporation and the degradation of volatile extractives from the fibres. During the second deterioration stage up to 331 °C, there was a significant mass loss of 32.41%. Hemicellulose expired between 184 and 331 °C. Cellulose and leftover hemicellulose were lost between 331 °C and 450 °C. Because of its complex design that incorporates aromatic rings with several branches, lignin generally decomposes slowly over the course of the entire temperature range up to 500 °C. Focusing on the breakdown of lignin and other non-cellulosic components found in the fibres, there was a final mass loss at 511 °C. The maximum temperature limit for untreated Zm root fibres is 525 °C, a value that is greater than the maximum temperature limit for KMnO_4_-treated Zm root fibres. A comparable decrease in the maximum temperature limit as a result of treatment was observed in jute and okra fibres. This was due to the lignin concentration of Zm fibres being decreased post-treatment. High thermal stability is correlated with a high lignin content. In the case of permanganate-treated Zm fibres, the temperature required to cause a 50% mass loss was higher. According to the chemical analysis of Zm fibres, this may be because the amount of hemicellulose being decreased after treatment. The sample that subsequently degrades to produce the ultimate remnant after the degradation is referred to as residual char^11,29,82^.

**Figure 9:**
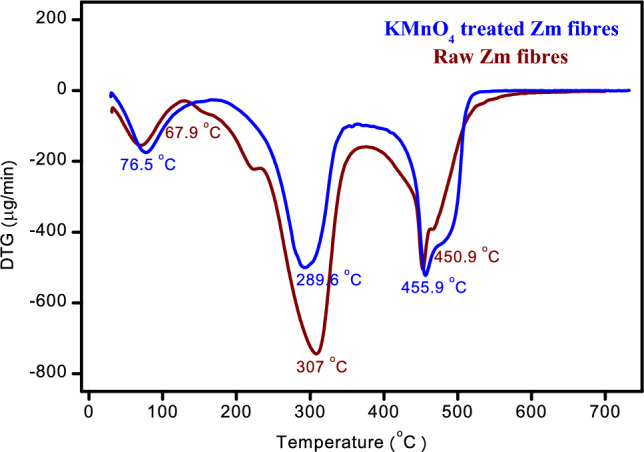
DTG curve of Zm root fibres.

The DTG curves of raw and permanganate-treated Zm fibres displaying the maximal breakdown rates for weight loss were shown in Fig. 9. Three peaks occurred at 76.5 °C, 289.6 °C, and 455.9 °C for treated fibres in the DTG scans that show the rate of weight loss (μg/min) vs temperature. The first peak is for the elimination of moisture content, while the second peak is for the decomposition of hemicellulose, and the third is for the approximate degradation of cellulose and lignin^2,29^.

#### Thermal activation energy

It is important from a practical standpoint to understand and forecast the thermal decomposition process of natural fibres. This information aids in improving the design of composite processes and can be used to estimate the impact of natural fibre in the thermal decomposition of composite materials^83^.

**Figure 10:**
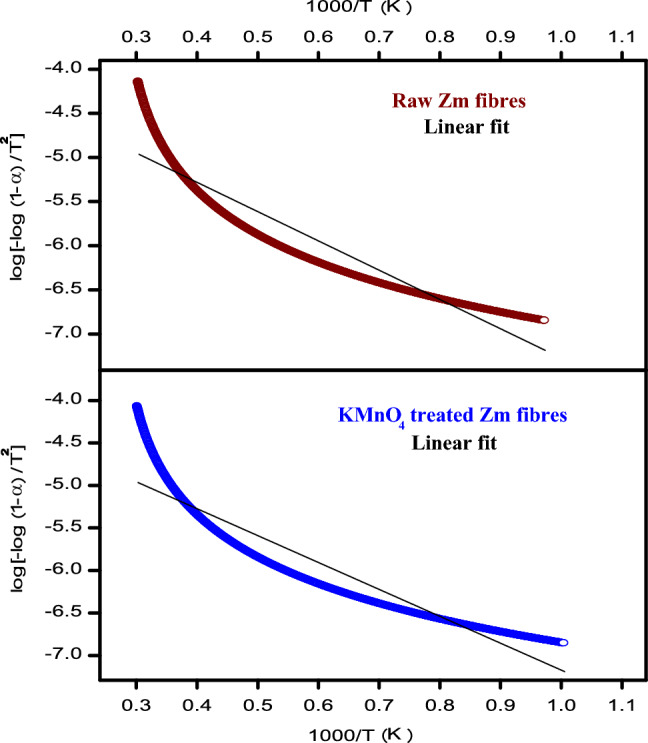
Coats—Redfern plot for Zm root fibres.

The apparent activation energy (E_a_) of untreated and treated Zm fibres was determined using the Coats-Redfern plot (Fig. 10). When compared to raw fibres, processed Zm root fibres used less energy in the decomposition process (Table 11). According to previously reported data, the range of activation energy needed for fibre breakdown in polymer composites is 60–170 kJ/mol^69^.

Activation energies of raw and permanganate treated *Zea mays* root fibres were found to be 63.48 kJ/mol and 60.58 kJ/mol. Kinetic activation energies of permanganate-treated *Butea parviflora* fibres (62.80–63.46 kJ/mol), *Lygeum spartum* (68.77 kJ/mol), Saharan aloe vera cactus (60.2 kJ/mol), and Indian areca fruit husk fibres (64.54 kJ/mol) were on a par with the value of permanganate treated Zm root fibres (60.58 kJ/mol). *Prosopis juliflora* bark (76.72 kJ/mol) and *Coccinia grandis* L. (73.43 kJ/mol) showed slightly higher activation energy values^29^.

#### Differential scanning calorimetry (DSC)

DSC analysis is used to estimate the heat generated or absorbed during chemical interactions among a fibre material's constituent elements when it is heated. Throughout the breakdown process, a variety of exothermic and endothermic processes occur at different temperatures. The endothermic and exothermic peak sizes and locations demonstrate the thermal phase transition of natural fibres. In contrast to endothermic events that induce heat absorption in the sample, exothermic events result in the release of heat.

**Figure 11:**
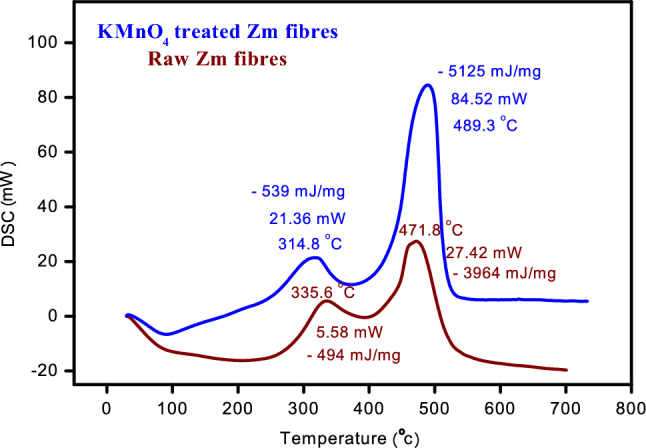
DSC curve of Zm root fibres.

Figure 11 shows the DSC profiles of Zm fibres treated with KMnO_4_ and those that have not been treated. Varying temperatures caused a large number of components to be lost. For both Zm fibre samples, there are two exothermic peaks. The peaks for raw sample are less intense when compared to the treated sample which shows the impression of permanganate treatment in Zm root fibres. The exothermic peaks at 314.8 °C and 335 °C indicates the combustion of hemicellulose and also other components found in Zm root fibres. The heat output in this case, however, was 539 mJ/mg at a consumption of about 21.36 mW energy for treated sample, while in raw sample it was 494 mJ/mg and 5.58 mW. The following exothermic peaks at 489.3 °C and 471.8 °C was due to the elimination of lignin and cellulose. At 489.3 °C, 5125 mJ/mg of heat was emitted by the treated Zm fibre sample, while 84.52 mW of energy was used. For raw sample, it was 3964 mJ/mg of heat and 27.42 mW of energy^23^.

### Elemental analysis

#### CHNS analysis

The CHNS investigation at (Table 8) below found that carbon was more abundant than other elements in both fibre samples. One of the most crucial components that could change the final product's mechanical and tribological properties is the high carbon content of natural fibres. In Zm root fibres, the hydrogen content was second after carbon (Table 8). Sulphur has been discovered in trace amounts in processed samples, but not in raw Zm fibre samples. Prior to treatment, the carbon concentration of Zm fibres was 41.84%; after treatment, the carbon content was 32.94%. In materials intended to lessen dielectric loss, both samples can be utilised as conductive fillers. Following potassium permanganate surface modification, the carbon content decreased, in accordance with the findings of the EDAX analysis^30^.

**Table 8:** CHNS analysis of Zm root fibres.

Sample	N%	C%	S%	H%	Sample weight (mg)
KMnO_**4**_-treated Zm root fibres	1.56	32.94	0.33	5.19	6.47
Raw Zm root fibres	1.76	41.84	ND	6.00	11.80

#### Energy dispersive spectroscopy (EDS)

**Figure 12:**
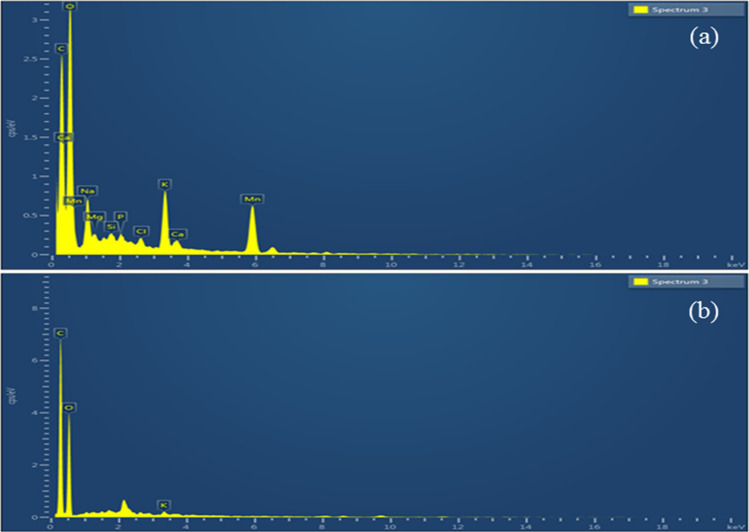
EDAX spectra of (**a**) KMnO_4_ treated Zm root fibres (**b**) Raw Zm root fibres.

Multiple elements found on the surface of Zm fibres can be identified with the aid of EDS. Figure 12 displays the EDS spectra of Zm fibres treated with permanganate and that of untreated fibres.

Elements on the surface, including their mass and atomic percentages of the permanganate-treated and raw Zm root fibres, were analysed using EDS. These fibres contained carbon and oxygen that could be expected from plant fibres. In addition, small proportions of potassium, sodium, chlorine, magnesium, manganese, silicon, phosphorus, and calcium were identified. Among the trace elements, the weight percentages of sodium, potassium, and manganese were high in the treated samples since the treatment was done using NaOH and KMnO_4_. Thus, the treatment's impact was evident. The carbon content after the treatment decreased, whereas the percentage of oxygen increased as mentioned in Table 9 below^4,23^.

**Table 9:** Weight and atomic percentages of elements present in Zm root fibres.

Elements	KMnO_4_-treated Zm root fibres		Raw Zm root fibres	
	Wt %	At%	Wt %	At%
C	38.62	49.69	55.99	63.03
O	44.94	43.41	43.55	36.81
K	3.48	1.38	0.46	0.16
Na	3.64	2.45	–	–
Mn	7.3	2.05	–	–
Mg	0.57	0.36	–	–
Si	0.25	0.14	–	–
P	0.36	0.18	–	–
Cl	0.43	0.19	–	–
Ca	0.4	0.15	–	–

Measuring the oxygen-to-carbon ratio in natural fibres is the goal of EDAX analysis. A higher carbon content indicates that the fibre contains more lignin. After being treated with permanganate, the carbon content declined and the oxygen content increased in Zm root fibres. A lower lignin was indicated by the higher O/C ratio. The O/C ratio can be used to explain a variety of behaviours of natural fibres. A lower O/C ratio suggests that fibres are more hydrophobic. Research on the behaviour of water absorption in samples of raw and permanganate-treated Zm root fibres supports this claim. Similarly, enhanced thermal stability was suggested by the decreased O/C ratio, as shown by the TGA-DTA analysis of Zm root fibres^84–86^. Table 10 below gives the O/C ratio of Zm fibres.

**Table 10:** O/C ratio of Zm root fibres.

Sample	Elements (At%)		O/C ratio	Lignin content (Wt%)
	O	C		
KMnO_4_-treated Zm root fibres	43.41	49.69	0.87	10.2
Raw Zm root fibres	36.81	63.03	0.58	19.04

### Scanning electron microscopy (SEM)

Whether the intended fibre may effectively serve as a reinforcing material depends critically on the surface morphology. The outermost layer of the fibre can be made rougher by chemical treatment that enhances the fibre's capacity to bond. Examining the fibre's morphology using scanning electron microscopy is the practical approach^23^.

**Figure 13:**
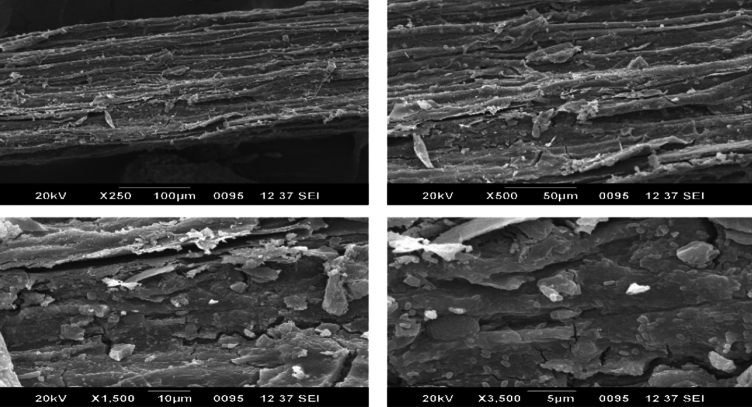
SEM images of permanganate-treated Zm fibres.

**Figure 14:**
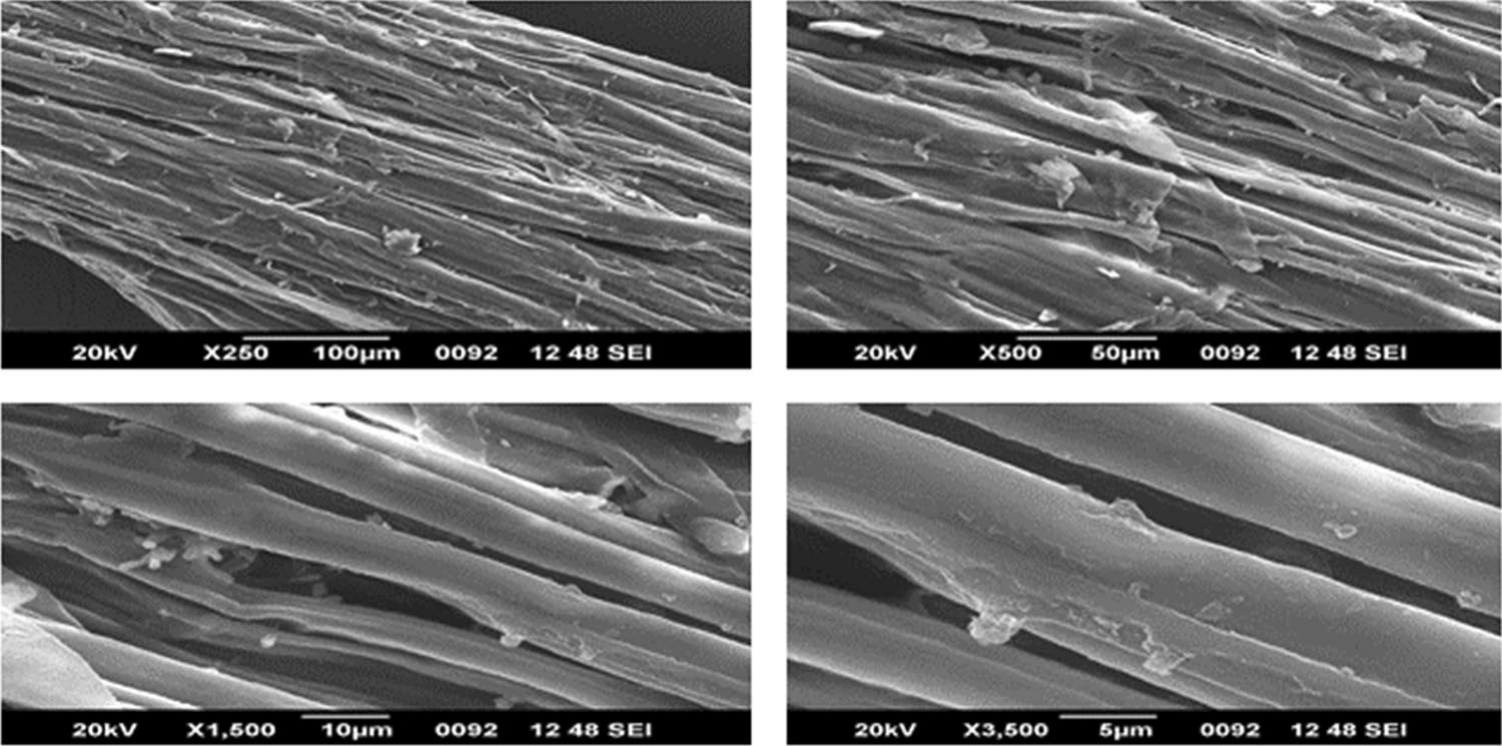
SEM images of raw Zm fibres.

The surface morphology of Zm fibres is depicted in Figs. 13 and 14. Raw Zm fibres have a distinct network structure, with hemicelluloses and lignin holding the fibrils together. Additionally, a protective layer was formed on the fibres' surface by wax, oil, and contaminants. These layers may result in weak interfacial adhesion between the polymer and the fibres. Rough and scaly structures with random particles dispersed over the exterior of the fibre were visible in the images of treated Zm fibres. Untreated Zm fibres’ primary cell walls disintegrate, causing the fibres to bend and decrease in diameter. The elimination of epidermis tissue that contains wax, lignin, and hemicellulose caused topographical alterations, and this was supported by the chemical analysis of Zm fibres. An increase in surface roughness may enhance the capacity of load transfer between the fibre and polymer as well as the interfacial connection between the two. Figure 14 illustrates the cylindrical shape and parallel arrangement of microfibrils that make up the Zm fibre. Furthermore, the treatment boosted the number of pits on the surface of the fibres.^54,71,79^.

Chemical processes clean the surface of the fibre, removing contaminants and non-cellulose elements. The structure, morphology, physical makeup, and chemical composition of natural fibres are all known to be altered once the fibres were treated with permanganate. To etch the Zm fibre surface, permanganate ions interact with the components of lignin. Additionally, the fibre surface was damaged by the treatment. The treatment damaged the cellulose structure and removed the majority of the hemicelluloses and lignin; this clarifies how the changes occurred^6,53^. Table 11 above compares the diameter of Zm fibres both prior to and after treatment.

**Table 11:** Diameter using ImageJ and Activation energy of Zm root fibres.

Sample	Diameter (µm)	Kinetic activation energy (kJ/mol)
KMnO_**4**_-treated Zm root fibres	255.595	60.58
Raw Zm root fibres	414.634	63.48

## Conclusion

The fibres from the roots of the corn plant *Zea mays*, which are normally regarded as waste, were analysed for potential use in composite materials. The physical, chemical, structural, spectroscopic, thermal, elemental, and morphological aspects of these fibres were examined. The alkali-pretreated Zm fibres were soaked in 0.1 M potassium permanganate solution for 10 min to modify their surface properties. Below is a list of all the differences in the qualities of the fibres before and after permanganate treatment.

1. According to the physical study of fibres, the treatment caused the density of the Zm fibres to increase from 0.7694 g/cm^3^ to 0.9241 g/cm^3^. After treatment, the thermal conductivity increased from 0.02990 W/mK to 0.04189 W/mK. There was a drop in aspect ratio (446.17 to 392.15). The linear density decreased (1509 tex to 707.26 tex). The diameter also decreased (345 µm to 448 µm). After permanganate treatment, the moisture absorption capacity was increased.
2. Chemical analysis revealed that following treatment, there was a decrease in the amounts of cellulose, hemicellulose, lignin, wax, and moisture. The amount of pectin and ash normally increased after treatment.
3. The p-XRD analysis indicated that the treatment caused the crystallinity to decrease (from 71.93 to 57.53%) while the crystallite size increased (1.45 to 2.2 nm).
4. Both fibre samples had similar peaks in their FTIR spectra; however, treatment had an impact on the peak intensity. Treatment with permanganate also caused some peaks to disappear.
5. TGA-DTA demonstrated that the treatment led to a decrease in the maximum temperature limit for Zm fibres (525 °C to 511 °C). For fibres treated with permanganate, the residual char at 700 °C was greater. The treatment caused the thermal activation energy to decline from 63.48 kJ/mol to 60.58 kJ/mol.
6. CHNS analysis indicated that the Zm root fibres had a high carbon content. According to the EDAX analysis, the surface of Zm fibres contained higher quantities of carbon and oxygen. Following treatment, there was a decrease in the carbon content in both elemental analyses.
7. SEM results showed that the surface got roughened by the permanganate treatment.

Based on these findings, it is reasonable to conclude that the permanganate treatment of Zm root fibres can have both positive and negative impacts when used as a reinforcing material in polymer composites. Both fibre samples could be used in a variety of applications where there is a high demand for natural fibre-reinforced composites, depending on the requirements of each application.

Making composites based on Zm root fibres and testing them for their properties are the future objectives of this research. Because of its chemical and environmental stability, high dielectric quality, and superior mechanical and adhesive properties, epoxy resin can be employed as the matrix material in the manufacture of natural fibre-reinforced polymer composites. Numerous alternative environmentally acceptable polymers, including bioplastics, natural rubber, and others, can also be utilised as a matrix, and their qualities can be compared. Further, the samples could be employed for Soxhlet extraction, and the fibre extract could then be employed immediately. The extract can be reprocessed into nanopowder and then employed as a reinforcing substance to obtain maximum usage of *Zea mays* root fibres.

## Data Availability

The datasets generated during and/or analysed during the current study are available from the author and corresponding author on reasonable request.
